# Assessment of myocardial motion in children and young adults using High-temporal resolution MR tissue phase mapping

**DOI:** 10.1186/1532-429X-16-S1-P328

**Published:** 2014-01-16

**Authors:** Keyur Parekh, Michael Markl, Patrick Magrath, Joshua D Robinson, Cynthia K Rigsby

**Affiliations:** 1 Northwestern University, Chicago, Illinois, USA

## Background

MR Tissue phase mapping (TPM) is a non-invasive tool to detect regional and global myocardial wall motion. Evaluation of myocardial velocity in radial and longitudinal direction throughout the cardiac cycle may help us better understand myocardial mechanics. Our goal is to investigate regional and global left ventricular myocardial motion using respiratory-gated high temporal resolution TPM in children and young adults.

## Methods

The HIPAA compliant prospective study was IRB approved. Twelve patients (mean age 14.8 years; range 5-22 years) underwent cardiac magnetic resonance (CMR) (1.5-T) including TPM (spatial resolution of 2.2 × 3.0 mm and temporal resolution of 20.4 ms). Nine patients with no intrinsic myocardial abnormality based on clinical history, standard biomarkers, MRI and echocardiographic data were compared with 2 patients having cardiac dysfunction and 1 patient with hypertrophic cardiomyopathy (HCM). Short axis images at base, mid-chamber and apex were manually contoured to outline the epicardium and endocardium. Data was post-processed using software programmed in Matlab (The Mathworks Inc., Natick, MA, USA) to obtain segmental radial and longitudinal velocities of left ventricle.

## Results

Regional and global assessment of radial (contraction and relaxation) and longitudinal (lengthening and shortening) myocardial motion could be detected. Peak radial and longitudinal velocity in systole and diastole were different in our age group compared to documented velocities in adults. Average peak longitudinal velocity in systole in HCM was 5.79 ± 1.82 cm/s (compared to 4.01 ± 2.33 cm/s in normals) implies hyperdynamic function in HCM. Average peak radial velocity in systole in patients with and without cardiac dysfunction was 2.71 ± 1.48 cm/s and 3.52 ± 1.81 cm/s respectively. Reduced velocities in patients with cardiac dysfunction indicate poor systolic function. Reduced average peak radial velocity in diastole in HCM (-3.82 ± 1.37 cm/s) compared to normal population (-5.93 ± 2.48 cm/s) denotes impaired relaxation of left ventricle and diastolic dysfunction in HCM.

## Conclusions

Velocity-mapping technique quantifies regional and global systolic and diastolic myocardial motion. Our preliminary result demonstrates a role for TPM to determine systolic and diastolic dysfunction in our age group. Myocardial velocities in children and young adults were different from documented velocities in adults. Recruitment is underway to validate our results in larger cohort.

## Funding

None.

**Figure 1:**
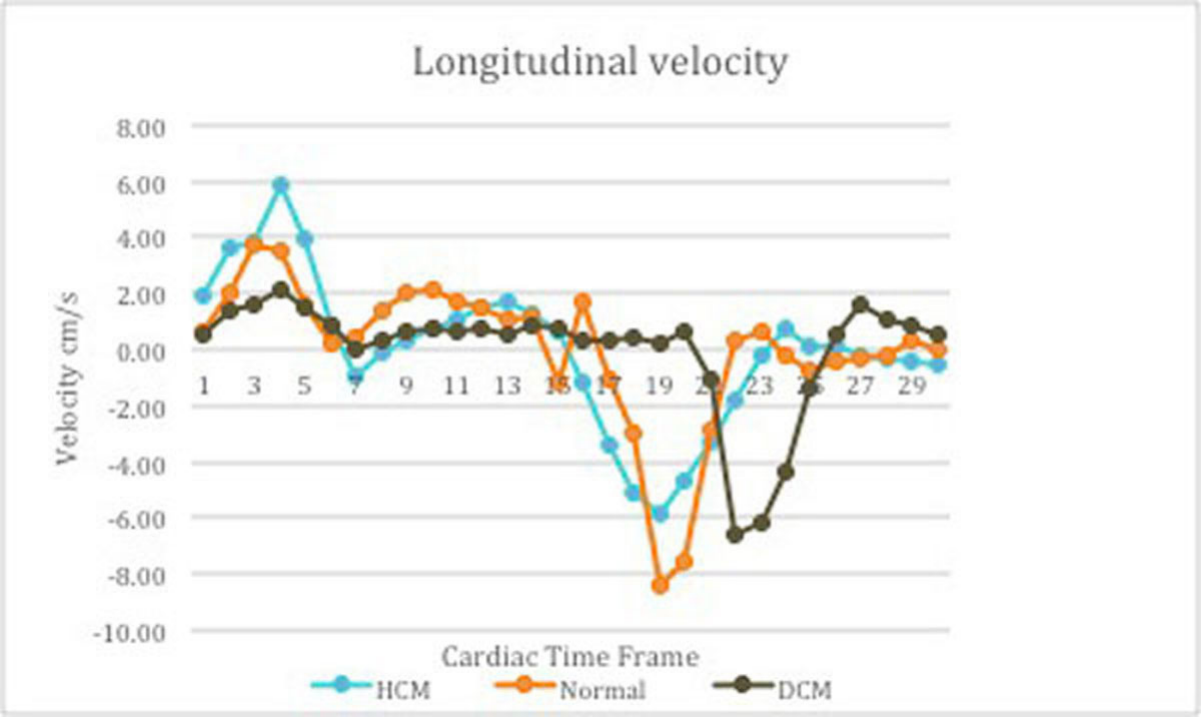
**Elevated systolic longitudinal velocity in hypertrophic cardiomyopathy (HCM) denotes hyperdynamic function and reduced velocity in patient with dilated cardiomyopathy (DCM) indicates systolic dysfunction**. Diastolic dysfunction in patients with HCM and DCM is demonstrated by reduced diastolic longitudinal velocities. Delayed relaxation of left ventricle in patient with DCM is noted.

**Figure 2:**
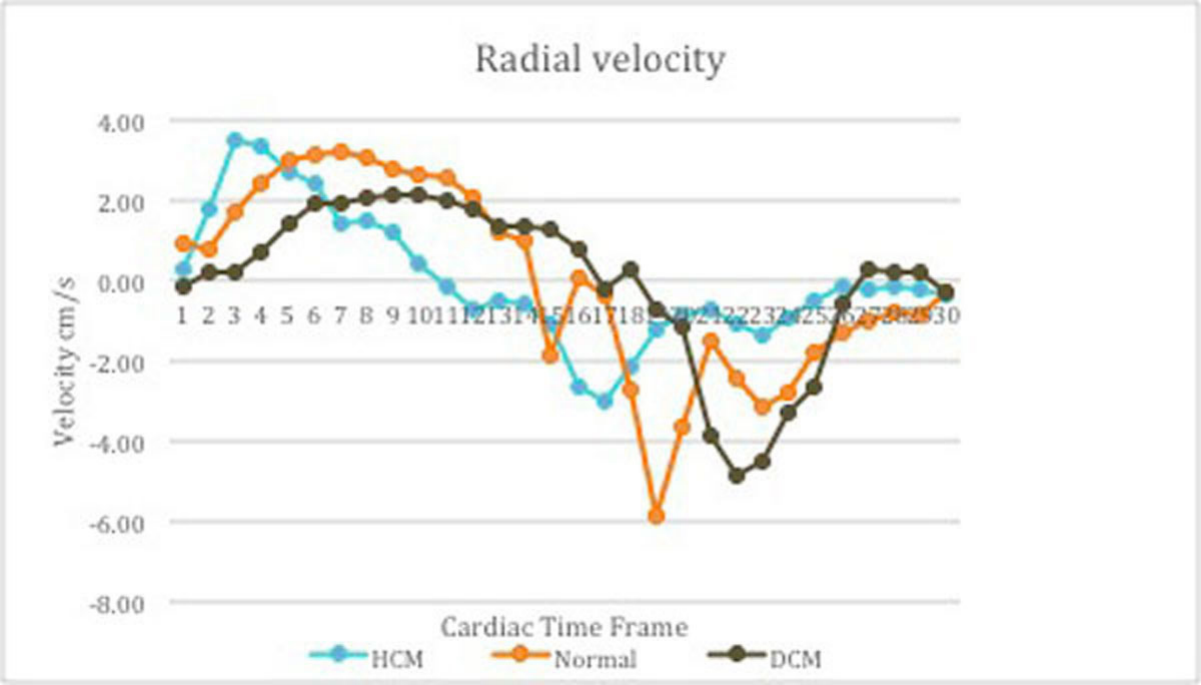
**Reduced time to peak during systole in hypertrophic cardiomyopathy (HCM) and sluggish peak with low velocities in patient with dilated cardiomyopathy (DCM) noted**. Impaired relaxation of left ventricle seen in both HCM and DCM patient.

